# Patient-provider interaction from the perspectives of type 2 diabetes patients in Muscat, Oman: a qualitative study

**DOI:** 10.1186/1472-6963-7-162

**Published:** 2007-10-09

**Authors:** Nadia Abdulhadi, Mohammed Al Shafaee, Solveig Freudenthal, Claes-Göran Östenson, Rolf Wahlström

**Affiliations:** 1 Department of Public Health Sciences, Division of International Health (IHCAR), Karolinska Institutet, SE-171 77, Sweden; 2 Department of Health Affairs, Ministry of Health, Oman; 3 Department of Family Medicine and Public Health, College of Medicine and Health Sciences, Sultan Qaboos University, Oman; 4 Skaraborg Institute for Research and Development, Sweden; 5 Department of Molecular Medicine and Surgery, Endocrine and Diabetes Unit, Karolinska Institutet, Sweden; 6 Department of Public Health and Caring Sciences, Uppsala University, Sweden

## Abstract

**Background:**

Patients' expectations and perceptions of the medical encounter and interactions are important tools in diabetes management. Some problems regarding the interaction during encounters may be related to a lack of communication skills on the part of either the physician or the patient.

This study aimed at exploring the perceptions of type 2 diabetes patients regarding the medical encounters and quality of interactions with their primary health-care providers.

**Methods:**

Four focus group discussions (two women and two men groups) were conducted among 27 purposively selected patients (13 men and 14 women) from six primary health-care centres in Muscat, Oman. Qualitative content analysis was applied.

**Results:**

The patients identified some weaknesses regarding the patient-provider communication like: unfriendly welcoming; interrupted consultation privacy; poor attention and eye contact; lack of encouraging the patients to ask questions on the providers' side; and inability to participate in medical dialogue or express concerns on the patients' side. Other barriers and difficulties related to issues of patient-centeredness, organization of diabetes clinics, health education and professional competency regarding diabetes care were also identified.

**Conclusion:**

The diabetes patients' experiences with the primary health-care providers showed dissatisfaction with the services. We suggest appropriate training for health-care providers with regard to diabetes care and developing of communication skills with emphasis on a patient-centred approach. An efficient use of available resources in diabetes clinics and distributing responsibilities between team members in close collaboration with patients and their families seems necessary. Further exploration of the providers' work situation and barriers to good interaction is needed. Our findings can help the policy makers in Oman, and countries with similar health systems, to improve the quality and organizational efficiency of diabetes care services.

## Background

Patients' perspectives and expectations are important elements in the patient-physician relationship regarding diabetes care [1]. If expectations are met during consultations, there is a positive association with patient satisfaction [2]. Health-care providers need to have good communication skills and relationships with diabetic patients to support their learning and to manage their illness in an appropriate way [3].

If diabetes care is to be delivered more effectively through general practice, there is need for efficient teamwork including diabetes specialist nurses, who have both the skill and time to address patients' needs within a consulting environment that respects the patients' own concerns [4]. It has been argued that physicians make limited efforts to foster patient involvement and autonomy that induce self-efficacy [5]. Enhancing the patients' active participation in diabetes care, and their self-care behaviour, is regarded to be a key factor to improve outcomes [6].

The Sultanate of Oman is located in the south-eastern portion of the Arabian Peninsula. The total population is 2.3 millions and approximately 632,000 live in the capital, Muscat. Forty percent of the Omani population are between 0–14 years, 56% are 15–64 years and 3% are 65 years or over. All Omani nationals enjoy free education through post-secondary school, vocational and higher education [7]. Some other demographic and social indicators are shown in Table 1.

**Table 1:** Demographic and socio-economic indicators in Oman

**Indicator**	**Estimate**
Total female population (%)	49
Literacy rate female/male	71/85
Population per one square kilometre	7.6
Life expectancy at birth (years) Female/Male	75/73
Crude death rate per 1000 Omani	2.7
Infant mortality rate per 1000 live births	10.3
Gross Domestic Product (GDP)	13.6
Total expenditure on health as percentage of GDP	3.2
Physicians' density per 1000 population	1.3
Nurses' density per 1000 population	3.5

Source: Final results of census 2003, Oman [7]

The health care system in Oman has been successful in controlling communicable diseases and childhood illnesses during the early 1990s and now has to face the challenges in controlling the non-communicable and lifestyle related diseases [8].

The rapid socio-economic development and social advances in Oman since 1970, has led to cultural changes including unhealthier eating habits, decreased physical activity and manifestation of a wide range of non-communicable diseases. Amongst them, type 2 diabetes mellitus is the most prevalent condition (11.6% in the year 2000), and becoming a growing health problem amongst Omanis [9]. Diabetes is more prevalent in the urban population (17.7%) than in the rural (10.5%) and crude estimates indicate that illiterate and less educated individuals in Oman are more likely to get diabetes [10].

The Ministry of Health has supported improvement in diabetes care through financial support and the development of detailed National Diabetes Guidelines for providers' performance in primary-care facilities, where diabetes care is mainly delivered [9]. To date, there are no published studies regarding patients' perceptions of the patient-provider interaction in Oman.

In this study, we used qualitative methods to explore the views and experiences of type 2 diabetic patients regarding the medical encounters with their primary health-care providers in Muscat in order to increase our understanding of the needs of type 2 diabetes patients in Oman and provide recommendations for the future care for these patients.

## Methods

This is an explorative study using focus group discussions (FGDs). It is part of a larger study including direct observations of doctors' and diabetes nurses' consultations and assessment of their performance [11], in-depth interviews with the health-care providers and collection of metabolic parameters. Those parts were conducted during 2004 in six primary health-care centres (PHCCs), with similar facilities for diabetes care.

Fifty-seven Omani patients with type 2 diabetes from both sexes, who attended the six PHCCs, were initially identified by the principal investigator, with the help of doctors and nurses in the health centres. The patients were purposively selected, using the criteria of having different educational levels, age groups, variations on diabetes duration and recruiting both men and women.

The selected patients were expected to participate actively in the discussions and provide useful information in terms of the study objectives. They were believed to constitute what Patton calls "information-rich" cases [12]. After being contacted, 42 patients agreed to participate, but finally only 27 patients (14 women and 13 men) were able to participate actively in the study. Eight patients were illiterate; seven had primary school education and twelve had intermediate to university level of education (details in table 2). Main reasons for declining as expressed by the patients were time constraints and social obligations. Some did not show up on the fixed dates for FGDs due to sudden illness or death of some members in the family. Decline could also be due to hesitation or other unknown reasons because the experience of conducting FGDs was new in Oman. The 30 patients who declined were similar to the participants in terms of demographic characteristics such as sex, age, and education level (details in table 2).

**Table 2:** Demographic characteristics of participants in the focus group discussions and of non-participants

	**FGDs 1 and 2**	**FGDs 3 and 4**	**Non-participants**
No. of participants	13	14	30
Sex	Men	Women	11 men, 19 women
Median age (range 26–70 years)	60 years	50 years	Men: 55 years Women: 50 years (range 25–70 yrs)
Illiterates	3	5	11
Primary education *(grade 1–6)*	4	3	7
Intermediate education *(grade 7–9)*	1	3	3
Secondary education *(grade 10–12*)	2	2	3
University	3	1	6

Four FGDs (two women and two men groups) with 6–8 participants in each group were conducted between July and August 2005. The participants agreed to conduct the FGDs in a meeting room in a secondary level health-care centre in Muscat, which they all perceived as a familiar and convenient venue and where they felt free to talk. Thematic guides for the FGDs were developed from the results of our previous observations [11]. Key areas explored included: patients' expectations, experiences, and views on the consultation environment and the provided care; experience with the diabetes nurses, dieticians and health educators; and recommendations for future improvement in the interaction and care (Appendix1).

The FGD sessions were led by an experienced moderator (the second author, who is a medical doctor and has experience in qualitative research methods, including using FGDs), who ensured that the discussion followed general recommendations [12,13]. The first author (a medical doctor, who has worked in primary care in Oman) took notes of the discussion and gathered information on the non-verbal communication and on the interaction between participants. The duration of the discussions was limited to two hours including around twenty minutes for greetings, warming up and introductory chat to create a relaxing atmosphere for all concerned. At the end of every focus group, there was a debriefing discussion between the moderator and the assistant moderator.

Each FGD was audio-tape recorded with the participants' consent, translated from Arabic into English language and transcribed verbatim by the first and second authors.

Qualitative content analysis was applied within the structure of the thematic guide and for the data that emerged from the materials [14]. The transcripts were read through several times by the authors (four medical doctors, where of two with experience of qualitative analysis, and one anthropologist) to obtain a good sense of the entire discussion. The text was then divided into meaning units and the meaning units were condensed. The condensed meaning units were then abstracted and labelled with codes independently by two of the researchers. The various codes were compared on the basis of differences and similarities and sorted into categories. The categories were further discussed by the authors for identification and formulation of themes and sub-themes. Quotations have been added to provide meaning to the text.

Ethical clearance and approval was obtained from the Medical Research and Ethics Committee of Oman. Verbal consents from the patients were obtained after explanation of the meaning of focus groups and the study objectives, and their anonymity was guaranteed.

## Results

Some of the patients' demographic characteristics are shown in Table 2. The participants had experiences with health-care providers of different nationalities including Arabic and non-Arabic speaking staff and many of them had experience from more than one health centre due to changes of the catchment areas or moving to another area.

Most of the participants talked to one another, commented on each others' experiences and points of view, asked questions during the sessions and raised issues related to the organization in diabetes clinics and access to care.

Six main themes were identified: 1) patient-provider communication manner, 2) inexperienced doctors and nurses, 3) long waiting time, 4) lack of continuity of care, 5) insufficient access to health education, 6) patient barriers to good diabetes management.

### Patient-provider communication manner

The patients discussed several topics that they considered as barriers for good patient-provider communication and friendly consultation environment such as: the manner in which they were greeted by the doctors and nurses; poor attention and eye contact during encounters; interrupted consultation privacy; lack of encouraging the patients to ask questions or express their concerns; and lack of transfer of medical information.

Both men and women expected to be welcomed cheerfully and with an introductory chat, but this was not done by all doctors and nurses regardless of their nationalities, cultural background or gender.

*Yes doctors greet us, only hello. They are not warm and not relaxed (man)*.

*I expect to meet a kind doctor who greets with a smile, asking me about my condition and if I sleep well or if I have any problems at home. This encourages me to express my concerns (woman)*.

*I am telling you the nurses never welcome the patient. They even irritate me and only raise my blood pressure (man)*.

Several patients expressed that some doctors seldom paid attention or looked at them during consultations, because they were busy writing the visit notes in the computers. In addition, they expressed that the use of computers during consultations consumes time at the expense of direct doctor-patient interaction.

*Doctors mainly look at the computers not at me. They are in another direction, they are not listening, or paying attention (man)*.

*The whole consultation time is not for us because most doctors write something in the computers and give us around five minutes (man)*.

A few women from one group had no negative views on the computers because they felt that the doctors listened to them attentively and looked at them while talking.

Some patients discussed the importance of privacy during encounters and mentioned that their privacy was interrupted during consultations with the doctors due to knocking on the doors and interruptions from other patients or colleagues.

*You may find more than one person in the doctor's room. The nurses bring patients to the room for blood pressure and sugar checking while I am still sitting with the doctor. What can I do in such a situation? (woman)*.

Most of the patients addressed the importance of the opportunity for patients to ask questions and express concerns. They added that doctors usually do not encourage them to ask questions during consultations or to participate in the medical dialogues, which make the encounters more doctor-centred and less friendly.

*Patient-doctor relation should be from heart to heart. I would feel happy if the doctor encouraged me to ask and talk freely. My sugar would also become good (woman)*.

*I am worried about my cholesterol level. They told me before in the hospital to be careful. But when I talked to the doctors in the health centre about it, they didn't care or showed just a good face listening to my worries. Doctors must listen (man)*.

Furthermore, both men and women were concerned about their blood test results and said they had not received feedback about them despite regular blood investigations. Some doctors provide the information upon patient request and others write the results in the patient's diabetes booklet without explanation, which was criticized because some patients are illiterate. In addition, most patients did not receive information about drug use and adverse events.

*The doctors never explained to me what is going on. Every time they take 5–7 bottles of blood and urine, but never tell what is wrong (man)*.

*I want to know more about some medicines, for example I am using Tenormin 50 mg, and they said it is for blood pressure but I don't have high blood pressure. Why don't they explain about medicines? (man)*.

In this context, it appeared from the discussions that language and cultural background of the health-care providers from other nationalities were not considered as barriers in the patient-provider communication and some patients acknowledged the positive manner of interaction among certain doctors from other nationalities.

### Inexperienced doctors and nurses

Many patients perceived the doctors and nurses in the diabetes clinics as not being experts and not competent enough in managing diabetes. Reasons for these perceptions were: brief consultations, infrequent physical examinations, doctors did not deal with diabetes as serious disease and did not consider their other health problems. Furthermore, it was expressed that there was poorer blood sugar control for the patients at the primary health-care level.

*Yes, they are not specialists, I knew this from the way they deal with us, only write the medicines and go, even not asking how we take it and when, and they don't examine the heart with that machine (stethoscope) (woman)*.

*Diabetes is not less serious than AIDS, but the primary-care doctors deal with it as common cold, If they found my weight and blood sugar OK they don't bother to ask about anything else (woman)*.

*I have a heart problem, this is my main concern. I complained to the doctors in diabetes clinic, they told me you have to speak to the cardiology doctor. Nobody cares. They don't look for the whole of problems (man)*.

The patients expressed that the role of diabetes nurses is very limited as they spend only 2–3 minutes with the patient whilst taking the basic measurements such as weight, blood pressures and blood sugar, but with no further communication or health education. Some of the diabetes nurses provide information only when asked.

*They don't talk to us, this is impossible. They check blood sugar and blood pressure, that's all (woman)*.

*The nurses are not experts. They do nothing. Doctors do everything (man)*.

### Long waiting time

Long waiting time up to four or five hours despite being given appointments was an issue that was raised spontaneously by almost all the patients and was expressed as stressful and unacceptable. However, some of the women considered long waiting time as a normal phenomenon in the PHCCs and felt it should not be an issue since they received free health services. In addition, they dealt with long waiting time by talking together or watching television in the waiting area. Reasons for the long waiting time in almost all the health centres as expressed by the patients were: only one doctor in the diabetes clinic; delay in the nurses rooms for check up of vital signs; disorganization from responsible staff regarding the queues; and patient factors such as not showing up on time for the given appointment.

*First of all no patient comes to the doctor unless there is something or according to appointment but when we come we are surprised that the doctor has to see a lot of patients may be 50, then it means if I come 8 am I have to see the doctor by 1 pm. This gives me stress and increases my blood pressure and blood sugar (man)*.

*Some doctors keep you waiting for 2 hours or more. I know the workers in the health centre do good things for us like starting by checking blood, taking some measurements and so, but still it is not logical to keep us waiting for all this long time, one day I waited four hours (woman)*.

### Lack of continuity of care

The patients mentioned that they see a different doctor every time. They said that the doctors differ in their behaviour and methods of providing care and information. Hence, they prefer to build an on-going relation with a certain doctor to avoid these differences.

*We don't meet the same doctor every time. I spoke to the doctor about my complaints and I highlighted the effect of some medicines on my body. He said give me time, I will study this and tell you next time what to do, but I don't know next time when I go if I will see the same doctor or not (man)*.

In this respect, there was no preference with regard to doctor's sex, except for one woman who preferred to be seen by a woman doctor for cultural reasons.

### Insufficient access to health education

Several patients had no interactions with the health educators or dieticians, irrespective of the duration of their diabetes. Some patients expressed that they had no idea about the availability of health educators or dieticians in the PHCCs or about their role as members in the diabetes team as recommended in the national guidelines and further clarified by the moderator during the discussions. The patients said that the health education was mainly provided by the doctors or through written educational materials, which were not considered to be useful by many patients due to their low literacy level. They addressed the need for continuous health education and the ability to support their learning by appropriate audio-visual aids especially in the waiting area. In a few cases, the patients had good experience of dieticians or health educators, but mainly during the first few months of diagnosis as they were not called for follow up.

*I have diabetes since ten years. Nobody referred me to a dietician or health educator. Doctors said my blood sugar is high. I asked them what to do, they only said take the medicine (man)*.

*I sat with a health educator long time when I started attending diabetes clinic but that was only once (women)*.

*Health educators? I never heard about them. May be not existing (man)*.

A few patients in this study had been educated about self-monitoring of blood sugar, but they expressed that they were unable to afford the blood glucose meters and test strips. In addition, many patients were not aware of the symptoms of hypoglycaemia and they asked the moderator for explanation. They expressed that the health care providers should give them such serious information even if the information was not solicited.

### Patient barriers to good diabetes management

Many patients reflected that they themselves could affect good diabetes management and patient-doctor communication. The patients said that they had poor compliance with diet control because they usually stick to their traditional food habits like eating dates in a frequent manner, and some families are not supportive to their diabetic members in terms of the way of cooking and preparing food. They addressed a need for education to family members and the community as well.

*The doctors are providing us with good health advice and we shouldn't blame them, we should blame ourselves. We are not following the advice because of our dietary habits and our families are not supportive. We find a lot of sweets like dates in the house and during social occasions and want to eat them. The diabetes patient should be the doctor of himself (man)*.

Low literacy amongst diabetes patients in Oman was perceived as another barrier for good diabetes management. A few women with low literacy levels believed that they had to accept what is provided to them because they are not educated. Hence, they felt unable to be more active during consultations. In addition, some thought that negotiations might negatively affect the interaction with health-care providers.

*Doctors only decide what to do because they know better. We don't know, we are not educated. We don't feel diabetes is a serious disease as (B) said, because we don't have symptoms (woman)*.

*Doctors treat us according to our behaviours, if we keep quiet they will treat us good, if we discuss, they may say these patients are primitive or problematic (woman)*.

This view was opposed by others, who were educated and younger in age.

*But if we discuss and ask or show our understanding and concerns, the doctors will respect us and treat us better (woman)*.

A similar view was also expressed by a few men who said that the doctors respect the educated patients or those who contribute actively to the conversation.

## Discussion

This explorative study identified a number of weak areas concerning patient-provider interactions and health care services in diabetes clinics in Muscat. The findings were strikingly similar in many aspects to other international findings regarding patient-provider interactions and health services in diabetes clinics at primary health care level. Weaknesses related to patient-provider communication were discussed such as the unfriendly welcoming, interrupted privacy during consultations and poor attention by the doctors due to use of computers during consultations. In addition, aspects related to patient-centred care and empowerment during doctors' encounters were discussed, such as lack of encouraging question asking and information transfer in particular with regard to blood investigations, information about medicines, hypoglycaemia and self-monitoring of blood glucose, but also patient barriers like traditional unhealthy food beliefs and low education among patients with diabetes. Furthermore, many patients questioned the competence of doctors and nurses in the diabetes clinics. Aspects related to access to care and organization of diabetes clinics were identified by both men and women as unnecessary long waiting time, lack of continuity of care and insufficient health education as they had limited experiences with the dieticians or health educators.

### Patient-provider communication

It has been concluded that unfriendly behaviour of health-care providers during medical encounters could be due to stress, work overload [15], and social inequalities between doctors and patients [16]. It was found in one interview study that encounters with professionals who were friendly and welcoming were considered as satisfying to patients with diabetes in primary health-care centres, while they described the dissatisfying encounters as being characterized by ignorance, including being treated unkindly or being made to feel unwelcome [17]. Moreover, attentive listening; eye contact with less gazes; uninterrupted consultation; and consultation lengths are important factors for a good patient-doctor communication and relationship [18,19]. Furthermore, in one study patients felt that they would not be bothered by computer use or writing during the patient interview if doctors continued to use verbal skills and maintain eye contact with them during consultations [20].

### Patient-centred care and adherence

Our findings reveal that the medical encounters in the health centers were characterized by more of physicians' dominance and less of attention to the patients' concerns, expectations and role in their own diabetes management and self-monitoring. It has been found that encouraging the patient to ask questions is not only a method of information seeking, but also a mechanism of patient participation in the medical dialogue which is positively associated with patients' satisfaction and health outcomes [21]. Furthermore, promoting the exchange of information between the doctor and the patient is a main purpose of medical communication and a facilitating mechanism for a patient-centred approach [22]. Patient-centred care is an interactive process between the doctor and patient and refers to seeking and accepting patient ideas, and giving recognition, encouragement, and sharing decision-making in response to the individual patient perspective [23]. However, organisational factors, such as workload pressure in the clinics and time allotted for the visits, may limit the propensity of health-care providers to adapt the patient-centred approach [24].

Researchers have concluded that patient participation in medical encounters depends on a complex interplay between a patient's personal characteristics such as gender, age and personality, cultural characteristics, and the physician's communication style [6,24,25]. Moreover, low education level among patients has been considered as a barrier for good communication and health outcomes due to its negative effect on patients' ability to communicate their history and on physicians' possibility to provide information [26].

It has been suggested that the ideal medical interview integrates the patient-centred and physician-centred approaches: the patients should lead in areas where they are the experts (symptoms, preferences, concerns); the doctors should lead in their domain of expertise (details of disease, treatment). However, before patients share decision-making power, they must first be offered by their doctors the choice of participation in the medical encounters and be provided with the medical information they need [22].

The low perception of some women in this study towards a patient-centred approach might not only be related to the social disempowerment of women in general as mentioned in some studies [27], it could also be related the power imbalances in the doctor-patient relationship, which can be manifested in doctors showing unconscious biases towards their patients and treating them according to their backgrounds. In doing so doctors may embed, in their interactions, forms of the 'inverse care law' where those most in need seem to get the least [16].

In a systematic review [6], it was concluded that a combination of approaches for improving diabetes patients' behaviour and enhancing their participation in the consultations, and a provider behaviour change of the consulting style into a more patient-centred one, possibly has got a considerable potential to produce better adherence and health outcomes. However, improving health outcomes of type 2 diabetes may not be possible without improving patients' self-management behaviour [6]. In particular, self-monitoring of blood glucose could considerably improve adherence to treatment and motivate patients to make appropriate life style changes [28]. Educational interventions could be useful for the health-care providers in Muscat, to promote a patient-centred approach during consultations and for the patients by providing structured and continuous health education to improve their perceptions, motivations and self-management and thereby their adherence to treatment recommendations. This area needs much attention and exploration in the Omani setting, especially as glucose meters and test strips might not be affordable for many people with diabetes.

### Culture, health care and organization

Patient-provider interaction is also affected by the cultural background of provider and patient. The influence of culture on the Omanis' behaviour and beliefs with regard to health issues and nutrition cannot be ignored. Religion is one of the dimensions of culture and social structure that affect the expressions, patterns, and practices of care within a culture [29], which must be seen in its particular context that is also made up of historical, ritual, family structure, food habits, social and geographical elements that mutually influence culture and are also influenced by culture [30]. Specific food habits are related to culture, such as consumption of dates served with coffee in Oman, which is a main delight that remains a symbol of Omani hospitality throughout the country. In Oman and in other Gulf states, dates might be taken frequently during the day. There is a strong cultural and religious belief about its nutritional and economic value, and it is considered as a blessing fruit. In smaller amounts dates are useful and nutritious, but the high sugar contents [31] make them unsuitable in larger amounts for diabetic patients. In general, there seem to be some misunderstandings and misbehaviour with regard to amount of food intake and to healthy nutrition in Oman. It should be mentioned that Islam considers health to be achieved only through taking good care of one's physical and mental health and taking every measure to maintain and enhance it, and that a healthy diet should be promoted, as well as not eating too much, with emphasis on wholesome food [32].

Changes in organization and relationships within the provider group and between providers and patients must also take the cultural context into account. In Oman, as in most Muslim countries, it is widely accepted that leaders will separate themselves from the general population, that there is a 'power distance' in the terms of Hofstede [33]. According to Hofstede there is also dimension of 'uncertainty avoidance' in Muslim countries, i.e., minimizing the possibility of ambiguity and uncomfortable situations. This is particularly prominent regarding the relation between doctor and patient, where the doctor is considered as a main source of security and knowledge and that the patients have to see the doctors and ask their help and advice because they have the power and they are responsible of their wellbeing. The concerns and suggestions expressed by the patients in this study, that they should be responsible of their own health and not depend too much on the doctors, may signal a shift to a situation where the patient is ready to take a more active role, although the cultural heritage will continue to influence the relationship.

These kinds of beliefs can have negative influence on the development of a more patient-centred care. The same is true for the dimension of 'individualism versus collectivism' [33] where Muslim societies, including Oman, are characterized by 'dependent collectivism' in which people from birth onwards are integrated into strong, cohesive in-groups, often extended families (with uncles, aunts and grandparents), which continue protecting them in exchange for unquestioning loyalty. This may be in conflict with the patient-centred approach, which has traditionally been of an individual character. The building of health care teams may therefore be particularly appropriate in the Omani context.

The health-care providers in diabetes clinics must be aware of the impact of culture and social context on peoples' behaviour, dietary beliefs and practices before these beliefs and practices can be modified or improved, as these elements play a role in decision making processes in the patients' everyday life with diabetes [6,30]

However, too broad generalizations in explaining people's beliefs and behaviours should be avoided, as there are other individually influencing factors such as age, gender, education (including education into a religious sub-culture), personality, intelligence, experience, occupation and socio-economic factors [30].

### Competence of doctors and nurses

Despite the availability and accessibility of health services in well-equipped diabetes clinics in Muscat, many patients expressed criticism of the provided services. They believed that the doctors and nurses were not experienced in managing diabetes. It has been found that good diabetes care with significantly better outcomes depends on the competency of the individual provider and the doctors' special interest in diabetes [34,35]. A comprehensive and integrated care should be provided to attain high quality management of diabetes and this includes the identification and treatment of risk factors associated with diabetes, such as micro-vascular complications and heart problems [36].

### Organizational efficiency and access to care

Patient satisfaction with waiting time plays a crucial role in the process of health quality assurance or quality management [37], and unnecessary waiting can be a cause of stress for both patients and doctors in general practice [38]. The combined effect of patients' adherence to actual appointment times, and lowering the patient:doctor ratio is important for reducing waiting times and improving the organizational efficiency of the diabetes services [39]. This approach could be implemented in the primary health-care settings in Oman.

The patients addressed the need for continuity of care with certain doctors. The concept of continuity of care can be described as a hierarchy ranging from availability of accurate information from one health care encounter to another (informational continuity), through a pattern of health care utilization at a particular site of care (longitudinal continuity), to an on-going personal doctor-patient relationship (interpersonal continuity) [40]. It has been concluded that continuity of care with a primary care provider is associated with better glucose control among patients with type 2 diabetes, and that this relationship appears to be mediated by changes in patient behaviour regarding food habits [41]. Moreover, interpersonal continuity with a certain physician seems to be important for the patient-doctor relationship and lead to the development of trust and confidence. Thus doctors and health-care managers should consider incorporating patients' preference for continuity into their office scheduling procedures [42].

Health education to diabetic patients is a necessary component of care and should be provided to the patients throughout and not only in the first few months of diagnosis. Health education should also be adjusted to the patient's own individual needs and could well be provided by diabetes nurses [43]. The nature of diabetes care requires a teamwork and diffusion of responsibility of care from physicians to nurses, dieticians, and further to patients and their families [44]. Most importantly, providers should base their health education on patients' unique understanding of their own situation [45]. It has been proposed that the appropriate use of simple and attractive visual tools during health education is effective and positively associated with health outcomes [46].

### Methodological considerations

The trustworthiness of the findings is essential in qualitative research. In this study we believe that we have reached a reasonable degree of credibility through the way in which we have conducted the interviews, including the questions asked. There was also a debriefing between the moderator (the second author) and the assistant moderator (the first author) at the end of every focus group to discuss the most important themes and possible differences with other focus groups. Furthermore, we have shown representative quotations from the transcribed text, which is regarded to enhance credibility.

Our decision to segment the FGDs by the participants' sex was based on two assumptions: a) in general, differences in perspectives between men and women may either reduce the comfort level in the discussion or affect how clearly either perspective gets discussed [13], b) specifically, despite the economic development in Oman and social advances for the women and their active participation in the development process all over the country, the social structure does not tolerate much of mixing between men and women, especially among less educated people.

We assessed it far better to avoid these expected constraints of mixing groups. Theoretically, it could be possible that some important aspects could have been brought up in a mixed group, but probably not in the present Omani context. Furthermore, the topic was general and there was no clearly gender related issues. A related aspect is that a man moderator conducted also the women FGDs, which could inhibit the discussion in contrast to if the moderator was from the same sex. However, the topics raised and the scope of the discussions were of similar character in the men and women groups, which may indicate that the field of exploration was not too sensitive to create uncomforting feelings among the women in the presence of a man moderator. In addition, the presence of certain young and educated women in the groups seemed to stimulate the others, who were less educated and might have been shyer.

In terms of dependability (truth value of results in relation to data) we think it is acceptably high in this study. We have mainly used the same question frame for all groups, although some new insights were acquired by the investigators that subsequently influenced follow-up questions or narrowed the focus for observation. Furthermore, judgments about similarities and differences of content were addressed by an open dialogue within the research team. Apart from debriefing between the first and second authors during the data collection phase, team members independently reviewed the transcripts and regular team meetings were held during data analysis to explore patients' underlying reasoning, to discuss deviant cases and to reach agreement on recurrent themes based on the pattern and relationship between the categories.

The usefulness or transferability of our results is dependent on how well we have been able to capture existing views and perceptions among patients with type 2 diabetes in primary care in Oman. One possible limitation could be related to the connection between the investigators and the authority or institution under study. Patients' fear of disclosure or fear of making revelations to members of their own social circle is also possible [47].

The strategy to select participants, who are expected to contribute 'rich information', may have some limitations [12]. The information available prior to selection may be inadequate and there might be a risk that the participants are selected too much on grounds of verbal competence. However, in this study we succeeded in recruiting participants with variation regarding education and diabetes duration.

There was some heterogeneity with regard to characteristics of some group members in terms of education level and age. The heterogeneity of the participants regarding their social background is known to have a potentially negative impact on the discussion [13]. However, in our study, this did not seem to reduce a productive sharing of essentially similar experiences [47].

Despite the constraints in recruiting more patients to the FGDs and the limited number of groups included in this study, we had rich information from the participants regarding the organization of the clinics and the manner of providers' interactions. The first and second authors were performing preliminary analysis all through data collection and felt that data saturation occurred, as they could no longer discern any new information. It is known that conducting more than 3–5 homogenous FGDs seldom provide meaningful new insight and additional data collection may no longer generate new understanding [13].

## Conclusion

We conclude that the diabetes patients' experiences with the primary health-care providers were articulated as dissatisfaction with the services in Muscat. The participants in this study reflected perceptions that are almost similar to what diabetic patients from Western countries have expressed.

Our study draws attention to some of the patients' concerns and preferences regarding delivery of diabetes services and patient-provider interaction and also to factors that underlie their preferences. We consider the patients' perceptions as important and that they should be taken in account to improve practice.

We suggest appropriate training for health-care providers regarding management of diabetes care and developing of communication skills with emphasis on a patient-centred approach and teamwork. Better utility of the available resources in the diabetes clinics and distributing responsibilities between team members in close collaboration with the patients and their families seems necessary. Further exploration of the providers' work situation and barriers to good interaction with patients is needed.

The findings of this study can serve as a point of departure for the policy makers in Oman and countries with similar health systems, for improving the quality of diabetes care, and for further improvement in the organizational efficiency of diabetes services.

## Appendix

### 1. Guide topics in the focus group discussions

1-We want you to discuss about your opinions and views on the interaction with the health-care providers and what you expect to get when you meet them during consultations? It is an open discussion and we want you to feel at ease and free to talk. We want to hear from all of you. We will start with the doctors, please tell us what you feel when you meet the doctors from the beginning of the consultation to the end of it, and what you like and what you do not like.

(Checklist for the moderator included: welcoming, consultation privacy, attention, eye contact, encouraging questions asking, and consultation length).

2-What is your opinion about the provided care?

(Checklist for moderator included: history taking, physical examination and role of the diabetes nurses).

3-Please tell us about your experience with the dieticians and health educators in your health centres?

4-A question for the females groups; How you perceive the encounters with male doctors?

5-What are your suggestions to improve the quality of interaction with the health-care providers?

## Abbreviations

FGD – Focus Group Discussion, MoH – Ministry of Health, PHCC – Primary Health Care Centre

## Competing interests

The author(s) declare that they have no competing interests.

## Authors' contributions

NA, MAS, CGO and RW contributed to the design of the study. MAS and NA conducted the FGDs, translated and transcribed them. SF together with NA, supported by MAS, CGO and RW analyzed the data and NA produced the first draft of the manuscript. All authors contributed to the write-up of the study, and have read and approved the final manuscript.

## Pre-publication history

The pre-publication history for this paper can be accessed here:


